# Modelling electrified railway signalling misoperations during extreme space weather events in the UK

**DOI:** 10.1038/s41598-024-51390-3

**Published:** 2024-01-18

**Authors:** Cameron J. Patterson, James A. Wild, Ciarán D. Beggan, Gemma S. Richardson, David H. Boteler

**Affiliations:** 1 Department of Physics, Lancaster University, Lancaster, LA1 4YW UK; 2 British Geological Survey, Edinburgh, EH14 4AP UK; 3 Natural Resources Canada, Ottawa, ON K1A 0E4 Canada

**Keywords:** Space physics, Natural hazards

## Abstract

Space weather has the potential to impact ground-based technologies on Earth, affecting many systems including railway signalling. This study uses a recently developed model to analyse the impact of geomagnetically induced currents on railway signalling systems in the United Kingdom during the March 1989 and October 2003 geomagnetic storms. The March 1989 storm is also scaled to estimate a 1-in-100 year and a 1-in-200 year extreme storm. Both the Glasgow to Edinburgh line, and the Preston to Lancaster section of the West Coast Main Line are modelled. No “right side” failures (when unoccupied sections appear occupied) are suggested to have occurred during either storm, and the total number of potential “wrong side” failures (when occupied sections appear clear) is low. However, the modelling indicates “right side” and “wrong side” failures are possible on both routes during the 1-in-100 year and 1-in-200 year extreme storms, with the Glasgow to Edinburgh line showing more total misoperations than the Preston to Lancaster section of the West Coast Main Line. A 1-in-100 year or 1-in-200 year extreme storm would result in misoperations over an extended period of time, with most occurring over a duration of 2–3 h either side of the peak of the storm.

## Introduction

Space weather can impact both ground-based and space-based infrastructures, potentially leading to communications disruption, power blackouts and damage to or interference with many systems. Of these impacts, geomagnetically induced currents (GICs) are considered one of the foremost concerns when considering the effects of space weather on ground systems. GICs arise when solar wind disturbances lead to fluctuations in ionospheric and magnetospheric currents systems, resulting in large and rapid magnetic field variations at the Earth’s surface. These magnetic field fluctuations generate geoelectric fields which cause induced currents to flow along extended conductors such as power grids^1–3^, oil and gas pipelines^4,5^, and railways^6–8^.

In July 1982, a geomagnetic storm caused railway signalling systems in Sweden to misoperate^9^. Signals were observed changing between green and red even though no trains were passing through the sections and no other faults could be determined. Research showed that the storm had generated a geoelectric field of $4-{5}\hbox {V}\,\hbox {km}^{-1}$, and GICs were identified as the cause of the signalling misoperation. Other studies of a statistical nature have demonstrated a correlation between an increase in the occurrence of railway signalling anomalies in Russia and heightened geomagnetic activity^10–12^.

Track circuits are the most common signalling system in the UK^13^, with more than 50,000 AC and DC track circuits being used across the network. In DC track circuits on AC-electrified lines, like those studied in this paper, one rail is continuous to allow for an unbroken return path to the power supply network for the currents that power the locomotives. This rail is called the “traction rail”. The other rail contains insulated rail joints (IRJs) that separate the line into individual blocks to allow for multiple signals to be placed. This rail is called the “signalling rail”. At the beginning of each block (in the direction of travel) is a relay that is connected via the rails to a power supply at the other end of the block. Both the relay and the power supply connect to the traction rail and the signalling rail, forming a complete circuit. Under normal conditions and with no train occupying the block, the current from the power supply energises the relay, causing the signal to be green. However, when a train enters the block, the conductive wheels and axles of the train redirect the flow of current such that the relay is de-energised, causing the signal to turn red. GICs can cause two distinct types of misoperation, described below. “Right side” failures are misoperations created when GICs cause the current through an energised relay (green signal) to drop below the level at which the relay will de-energise (drop-out current), turning the signal red and making it seem like there is a train in that section when there is not. “Wrong side” failures are a hazardous case when GICs drive the current through a de-energised relay in an occupied block (red signal) above the level to re-energise the relay (pick-up current), turning the signal green and causing the block to appear clear, giving no warning for approaching trains to stop.

**Figure 1:**
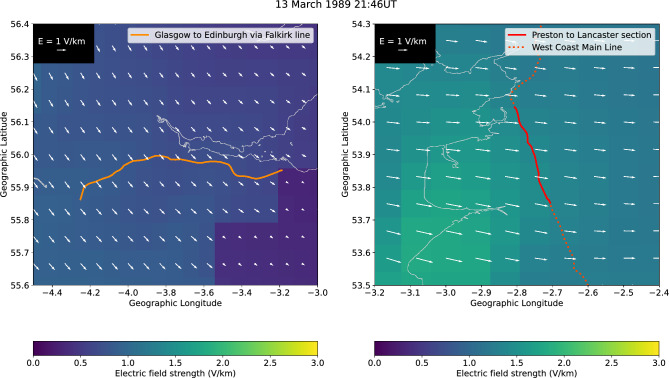
Geographic maps of sections of the UK surrounding the Glasgow to Edinburgh via Falkirk line (left) and the Preston to Lancaster section of the West Coast Main Line (right). The underlying colours give the magnitude of the geoelectric field in each grid cell for a peak value of the March 1989 storm on 13 March 1989 at 21:46UT, while the white vectors give the direction of the geoelectric field. The lengths of the white vectors also indicate the magnitude of the geoelectric field, with a scale given in the top left of both panels.

**Figure 2:**
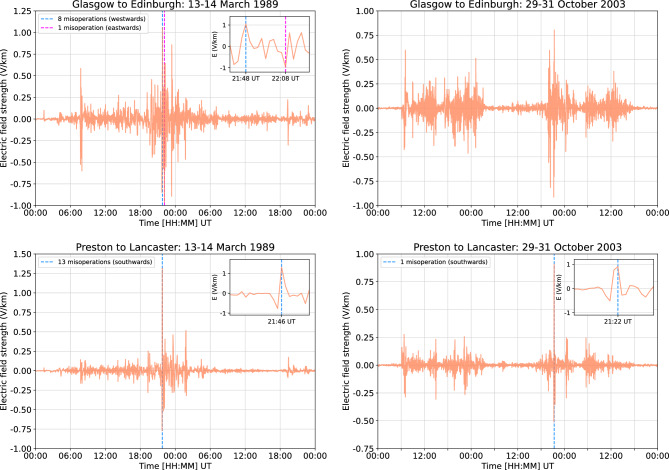
The electric field parallel to the rails near the centre of the Glasgow to Edinburgh line and the Preston to Lancaster section of the West Coast Main Line during the March 1989 and October 2003 storms. The Glasgow to Edinburgh line is generally east–west orientated, and the Preston to Lancaster section of the West Coast Main Line is generally north-south orientated. The dashed blue and red lines show the times that misoperations occurred on those lines, with the total number of misoperations and the direction of travel they occurred in shown in the top left. The smaller panels in the top right show the same data but for a shorter time period, allowing for closer examination of the features within that region.

In this study, we use a recently developed model^14,15^, based on earlier theoretical work^8^ and adapted for the UK, to analyse the impacts of GICs on two UK railway network routes: the generally east–west orientated Glasgow to Edinburgh via Falkirk line and the north-south orientated Preston to Lancaster section of the West Coast Main Line (WCML), both shown in Fig. 1. Firstly the model is used to estimate misoperations that could have occurred during the March 1989 and October 2003 storms, using electric field data generated from a thin-sheet conductivity model covering the UK^16,17^. The strength of the electric field parallel to the rails for both storms near the middle of the Glasgow to Edinburgh line and the Preston to Lancaster section of the WCML are shown in Fig. 2.The component of the electric field parallel to the blocks was extracted from these values. Secondly, the March 1989 storm is scaled by a factor of 2 to estimate a 1-in-100 year extreme storm and by 4 for a 1-in-200 year extreme storm. The same analysis is then performed for these extreme storm estimates. Finally, the timing of the misoperations is examined to determine the duration in which misoperations might occur during the extreme storm estimates.

The results presented here are a first version of the analysis of the impacts of geomagnetically induced currents on railway signalling systems using more realistic variations of the geoelectric field rather than the simple uniform field in previous studies^14,15^. This will be refined as the measurement and modelling of the geoelectric field and the rail network are improved over the next few years.

## Results

### Historic storms

Two of the largest geomagnetic storms in the past 40 years were analysed in this study: 13–14 March 1989 and 29–31 October 2003. The March 1989 storm caused large GICs to be generated within the Hydro-Québec power system in Canada, which led to a blackout that left affected areas without power for several hours and caused widespread effects on power systems in North America and northern Europe^18,19^. The October 2003 storm caused issues in the power network in southern Sweden, and was responsible for the loss of the $640 million ADEOS-II satellite^20^. Storms on 24 November 2003 and 7–8 September 2017 were also modelled but showed no misoperations, so they are not examined further. These storms were chosen due to them being the largest and most notable in terms of impacts in recent decades.

**Figure 3:**
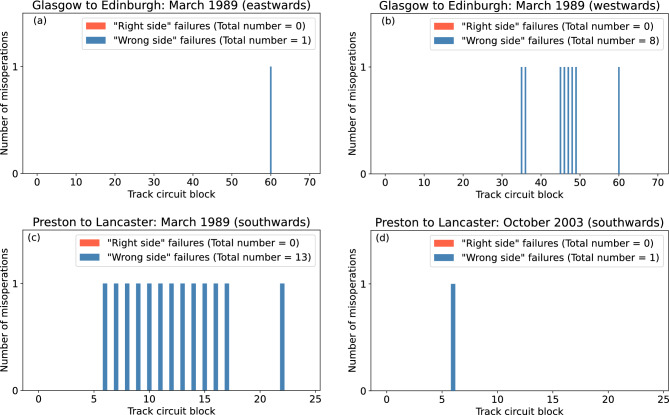
The total number of signal misoperations along the Glasgow to Edinburgh line during (**a**) the March 1989 storm in the eastwards direction of travel, and (**b**) the March 1989 storm in the westwards direction of travel. Glasgow is at block 0 for both directions of travel. The results for the Preston to Lancaster section of the WCML are also shown for during (**c**) the March 1989 storm in the southwards direction of travel, and (**d**) the October 2003 storm in the southwards direction of travel. Preston is at block 0 for both directions of travel.

The total numbers of “right side” and “wrong side” failures in all of the 70 track circuit blocks in the Glasgow to Edinburgh line for the duration of each storm were modelled. The results suggest that no “right side” failures would have occurred in either direction of travel during either storm. Figure 3 shows that for the March 1989 storm, there would have been the potential for 1 “wrong side” failure in the eastwards direction of travel (Fig. 3a), occurring at 13 March 1989 22:08UT, and 8 in the westwards direction of travel (Fig. 3b), occurring at 13 March 1989 21:48UT. For the October 2003 storm, the model indicates that no “wrong side” failures would have occurred in either direction of travel.

This analysis was repeated for the Preston to Lancaster section of the WCML. Similar to the Glasgow to Edinburgh line, the model suggests that no “right side” failures would have occurred in any of the 25 track circuit blocks in either direction of travel during both storms. For the March 1989 storm, there would have been no “wrong side” failures in the northwards direction of travel and 13 “wrong side” failures in the southwards direction of travel (Fig. 3c), occurring at 13 March 1989 21:46UT. For the October 2003 storm, the model suggests that no “wrong side” failures would have occurred in the northwards direction of travel and a single “wrong side” failure in the southwards direction of travel (Fig. 3d), occurring at 30 October 2003 21:22UT.

### Extreme storms

**Figure 4:**
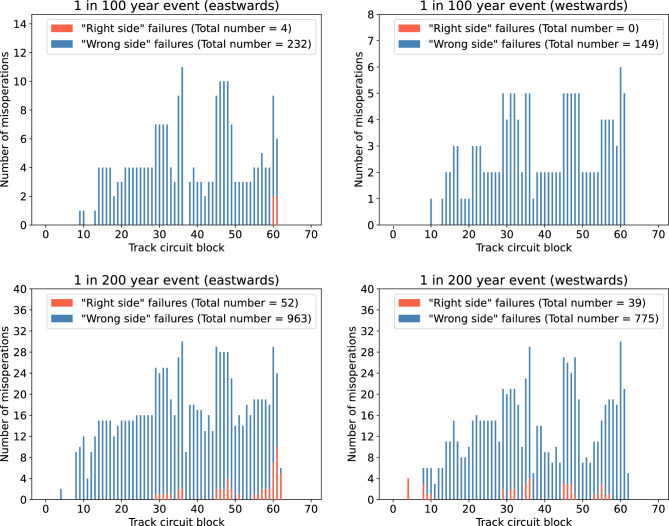
Glasgow to Edinburgh: the total number of signal misoperations in each track circuit block in the eastwards direction of travel and the westwards direction of travel during a 1-in-100 year extreme event and a 1-in-200 year extreme event.

The geoelectric field values estimated for the March 1989 storm were scaled up by a factor of 2 to provide a time-varying example of the electric fields during a 1-in-100 year extreme geomagnetic storm, and a factor of 4 for a 1-in-200 year extreme geomagnetic storm. These values were then input into the model to provide an estimate for the number of “right side” and “wrong side” failures we might expect to see during storms of these magnitudes. Figure 4 shows the total number of both misoperation types for the Glasgow to Edinburgh line. Throughout the 1-in-100 year extreme storm, the model suggests there would be 4 “right side” failures and 232 “wrong side” failures in the eastwards direction of travel, and no “right side” failures and 149 “wrong side” failures in the westwards direction of travel. For the 1-in-200 year extreme storm, the number of misoperations increases to 52 “right side” failures and 963 “wrong side” failures in the eastwards direction of travel, and 39 “right side” failures and 775 “wrong side” failures in the westwards direction of travel.

**Figure 5:**
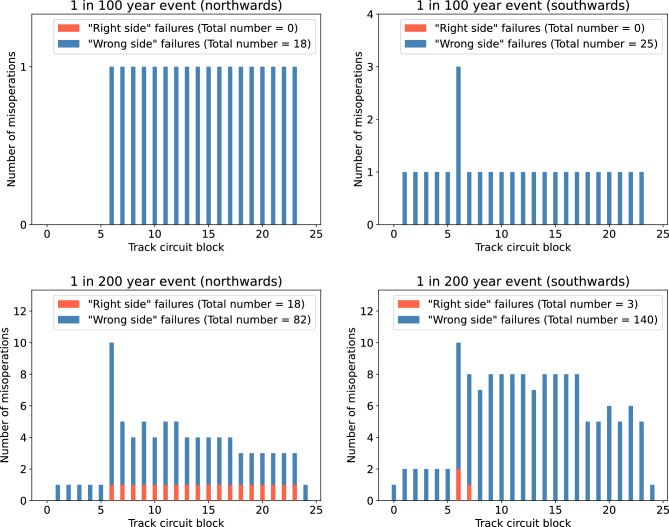
Preston to Lancaster: the total number of signal misoperations in each track circuit block in the northwards direction of travel and the southwards direction of travel during a 1-in-100 year extreme event and a 1-in-200 year extreme event.

Figure 5 shows the total number of both misoperation types for the Preston to Lancaster section of the WCML. For the 1-in-100 year extreme storm, the model suggests there would be no “right side” failures in either direction of travel, however there is the potential for 18 “wrong side” failures in the northwards direction of travel, and 25 “wrong side” failures in the southwards direction of travel. For the 1-in-200 year extreme storm, the model suggests there would be 18 “right side” failures and 82 “wrong side” failures in the northwards direction of travel, and 3 “right side” failures and 140 “wrong side” failures in the southwards direction of travel.

### Time series analysis

**Figure 6:**
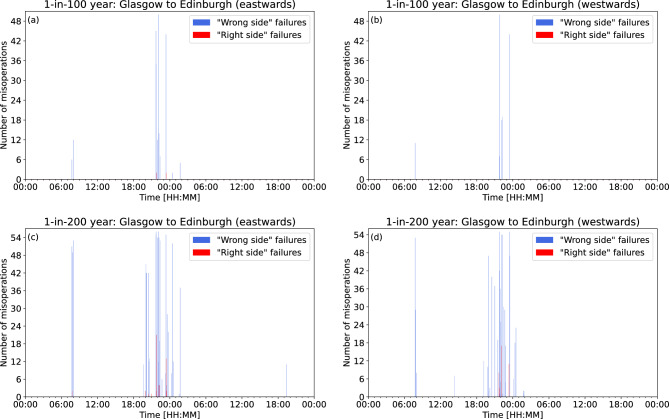
Glasgow to Edinburgh: the total number of signal misoperations at each 2-minute interval in (**a**) the eastwards direction of travel and (**b**) the westwards direction of travel during a 1-in-100 year extreme event, and in (**c**) the eastwards direction of travel and (**d**) the westwards direction of travel during a 1-in-200 year extreme event.

Considering the number of misoperations by when they occurred rather than in which block (as in Fig. 3), the temporal spread of misoperations throughout the storm can be analysed. Note that UT is close to magnetic local time in the UK. Focusing on the extreme storm estimates, Fig. 6 shows the total number of “right side” and “wrong side” failures for each 2-minute time period that the data covers for the Glasgow to Edinburgh line. For the 1-in-100 year extreme storm, “right side” failures are expected to occur just before 22:00UT and shortly after 23:00UT in the eastwards direction of travel (Fig. 6a), with only a couple of occurrences at each time, while no “right side” failures occur in the westwards direction of travel (Fig. 6b). “Wrong side” failures are shown to occur in smaller numbers around 08:00UT and more frequently 21:00UT and 02:00UT in both the eastwards and westwards directions of travel. For the 1-in-200 year extreme storm, there are now instances of “right side” failures occurring in both the eastwards direction of travel (Fig. 6c) and westwards direction of travel (Fig. 6d). In the eastwards direction of travel, there are now “right side” failures at 08:00UT and an increased number of “right side” failures between 20:00UT and 00:00UT. “Wrong side” failures have the potential to occur far more often during the 1-in-200 year extreme storm than the 1-in-100 year extreme storm, and the period they occur in has expanded to between 19:00UT and 02:00UT, with new occurrences around 14:00UT in the westwards direction of travel.

**Figure 7:**
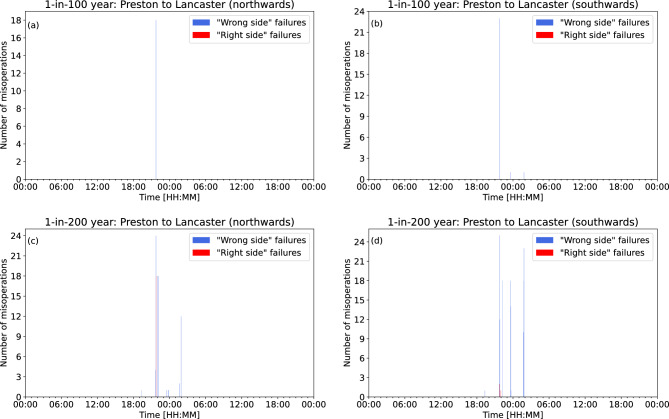
Preston to Lancaster: the total number of signal misoperations at each 2-min interval in (**a**) the northwards direction of travel and (**b**) the southwards direction of travel during a 1-in-100 year extreme event, and in (**c**) the northwards direction of travel and (**d**) the southwards direction of travel during a 1-in-200 year extreme event.

The results for the Preston to Lancaster section of the WCML are shown in Fig. 7. For the 1-in-100 year extreme storm, there are no “right side” failures northwards or southwards directions of travel. There are “wrong side” failures just prior to 22:00UT in both directions of travel, and some around 23:30UT and 02:00UT in the southwards direction of travel. For the 1-in-200 year extreme storm, there are “right side” failures in both directions of travel at around 22:00UT. “Wrong side” failures generally occur just after 19:00UT, around 22:00UT, between 23:00UT and 00:00UT, and just before 02:00UT.

## Discussion

The model suggests that for the March 1989 storm, there was the possibility for a number of “wrong side” failures to have occurred, mostly in the westwards and southwards directions of travel for each line respectively, as shown in Fig. 3. This is due to the peak strength of the electric field parallel to the rails being larger in the positive direction than the negative direction, as shown in Fig. 2, and a positive electric field drives “wrong side” failures in the westwards and southwards directions of travel^15^. For the October 2003 storm, the model suggests that there were no misoperations on the Glasgow to Edinburgh line, and only the potential for a single “wrong side” failure on the Preston to Lancaster section of the WCML, occurring in block 6. This block is the longest in the Preston to Lancaster section of the WCML, and thus is the most susceptible to misoperation^14^. Also, the positive peak of the northwards electric fields of the October 2003 storm was larger than the negative, which is why the “wrong side” failures occur in the southwards direction of travel but not the northwards direction of travel. The model suggests that for both sections, no “right side” failures would have occurred during either of the storms analysed in the study. It is expected that fewer “right side” failures would occur than “wrong side” failures, as the electric field strength required for “right side” failures to occur is higher than for “wrong side” failures^15^. It is important to note that “wrong side” failures can only occur when a train is occupying a block, and the strength of the geoelectric field needed to cause a misoperation is dependent on the distance the train has travelled through said block^15^. Therefore, even though there is the potential for a “wrong side” failure, it does not equate to a definite misoperation. It is difficult to validate whether misoperations actually occurred, as records of railway anomalies are often not digitised and since misoperations caused by space weather do not damage equipment, events may go undocumented. This highlights the importance for railway operators and engineers to log all anomalies, even those that seem to have no obvious cause.

During a 1-in-100 year and 1-in-200 year extreme storm, the Glasgow to Edinburgh line could experience both misoperation types, as shown in Fig. 4. The number of “right side” failures falls short of the number of potential “wrong side” failures, which indicates that storms of this magnitude have the potential to both cause disruption and lead to a hazardous situation. Figure 5 shows the results for the Preston to Lancaster section of the WCML, with the total number of misoperations being far fewer than for the Glasgow to Edinburgh line. This is not only due to the respective length of each line and corresponding number of blocks, but also because the electric field is on average weaker in more southern locations. This is demonstrated in Fig. 2, where even though the peak positive electric field during the March 1989 storm is larger at the Preston to Lancaster section of the WCML than at the Glasgow to Edinburgh line, the other fluctuations on either side of the peak are larger overall at the Glasgow to Edinburgh line. It can be seen that for the Glasgow to Edinburgh line, the misoperations seem to not impact blocks at the start and end of the line, but within the Preston to Lancaster section of the WCML there are misoperations throughout. This difference is due to the Glasgow to Edinburgh line being a complete line and blocks at the end of the line are more resilient to the impacts of GICs^14^, whereas Preston to Lancaster is only a section of the longer WCML, and so the start and end of that section are not truly the ends of the line. It should be acknowledged that differences in the geomagnetic storm signal can lead to significant differences in the number of misoperations observed. A future study could examine the impact of this, perhaps by means of superposed epoch analysis. Since the thin-sheet model is known to underestimate geoelectric field strengths, there is further motivation for a more detailed future investigation, as with larger electric field strengths, the potential for signal misoperations is higher.

One source of variability in these results is the rails’ leakage, which can change depending on environmental conditions, increasing with wetter weather and decreasing with drier weather. These changes impact how susceptible the track circuits are to GICs, with the potential for misoperation being higher in wetter conditions and vice-versa. While in this study we have assumed moderate leakage, the analysis was repeated for both wet and dry conditions (given in Table 1). As expected, the model suggested a higher number of misoperations could occur during wetter conditions, including cases where it was previously suggested there would be no misoperations. In drier conditions, the total number of misoperations that could occur decreased. For example, no “right side” failures were suggested to occur on either route during both the March 1989 and October 2003 storms under moderate leakage conditions. However, under maximum leakage conditions, the total number of “right side” failures suggested to occur during both storms on both routes increased to 90. For the 1-in-100 year extreme storm, at maximum leakage conditions, the total number of “right side” failures across both routes increased from 4 to 503; for the 1-in-200 year extreme storm, there was an increase from 112 to 2194. As the threshold for “wrong side failure” is not as impacted by changes to leakage, it is expected that while there would be some increase, it would not be as significant as the “right side” failures results^15^.

The number of misoperations over the course of a 1-in-100 year and 1-in-200 year extreme storm for both the Glasgow to Edinburgh line and the Preston to Lancaster section of the WCML are shown in Figs. 6 and 7. Most misoperations in the extreme events considered here occur within a few hours before and after local midnight. This is because the main phase of the 1989 storm (on which the extreme storms are based) peaked just prior to midnight on 14 March, with the Disturbance Storm-Time (Dst) index approaching -600 nT at that time and magnetic disturbances in the UK peaking in the hours that followed due to the southward expansion of the auroral oval and associated electrojets to mid-latitudes^19^. In this scenario, most misoperations would occur outside of peak railway operating hours. Also, since there are fewer trains on the line during those periods, the probability of a “wrong side” failure arising is lower. However, geomagnetic disturbances at any particular place will vary with local time as the Earth’s rotation carries that location through the disturbed region on the night side of the earth. This means storms can occur at any time, and could last for multiple days. Thus, greater consideration should be given to the total duration of the spread of the misoperations, rather than the specific time itself. The model suggests that for both the Glasgow to Edinburgh line and the Preston to Lancaster section of the WCML, there are short periods where multiple misoperations could occur, separated by periods of no misoperations. Misoperations could potentially impact signaling systems over a period of 2–3 h on either side of the peak of the storm. This prolonged disruption to the network could cause delays and increases the potential for a hazardous situation to occur. Though no storm has the same profile as another, our modelling suggests that an extreme geomagnetic storm has the potential to impact UK railway signalling systems.

## Methods

### Track circuit modelling

The rail model used in this study, adapted for the UK from theory described in Boteler^8^ and first demonstrated in Patterson et al.^14^, calculates the current through track circuit relays within a railway line under varying applied electric field values and compares them to the configured current levels of the relay to determine whether signalling systems are misoperating. The Glasgow to Edinburgh line consists of 70 blocks of $0.4-{1.9}\,\hbox {km}$ in length, with the total traction rail length of around ${76}\,\hbox {km}$. The Preston to Lancaster section of the WCML is split into 25 blocks of $0.8-{1.6}\,\hbox {km}$ totalling about ${34}\,\hbox {km}$ in length, the total traction rail length is unspecified as this is a section of a longer line.

Each rail was modelled as a transmission line, with series impedance determined by the rail’s resistance and parallel admittance by its leakage to the ground. The transmission line of each rail was then split and transformed into multiple individual equivalent-pi circuits, each corresponding to a single block. The equivalent-pi circuits of adjacent blocks were then merged, consolidating the connection points into a series of individual nodes. Power supply and relay components were then introduced, with values sourced from Network Rail standards and given here in Table 1, which connected both rails and completed the basic nodal network. The nodal network was then expanded with the addition of cross bonds, wires that connect the traction rails of multiple tracks together, with each cross bond adding one new node on each of the corresponding traction rails. If trains were to be modelled on the line, the multiple axles of the train were added. Each axle was the equivalent of a low resistance connection between the signalling and traction rails, which added one node on each rail for every axle. The network’s impedance values were then converted into admittances and used to form the nodal admittance matrix [*Y*], which had size $N\times N$, where *N* was equal to the total number of nodes in the network. Within this matrix, the diagonal elements were the total sum of admittances into the nodes with indices matching the row and column indices, while off-diagonal terms were the negative of the admittance between nodes. The admittances between each pair of nodes that have no connection, for example, where the IRJs separate the signalling rails, are set to zero. The electric field along the line was then added, modelled as separate voltage sources distributed between the nodes of each block. As the direction of the electric field changes with time, the component parallel to the rails will vary. As such, it is worth noting that the Glasgow to Edinburgh line is generally east–west orientated, while Preston to Lancaster is north–south orientated. These voltage sources were then transformed into equivalent current sources, as detailed below.

The power supply equivalent current sources, $I_{power}$, were calculated using Eq. (1), where $V_{power}$ was the voltage of the power supply and $r_{power}$ was the resistance of the power supply’s accompanying resistor.

1 $$\begin{aligned} I_{power}=\frac{V_{power}}{r_{power}} \end{aligned}$$

The electric field equivalent current sources along the line were calculated with Eq. (2), where $E_{\parallel }$ was the parallel electric field component to the rail and *Z* was the series impedance per unit length of the rail.

2 $$\begin{aligned} I_{E}=\frac{E_{\parallel }}{Z} \end{aligned}$$

The total sum of equivalent current sources directed into each node were calculated to form a matrix of nodal equivalent current sources, [*J*]. Equation (3) shows the relationship between [*J*], the nodal voltages [*V*], and the network admittances [*Y*].

3 $$\begin{aligned}{}[J]=[Y][V] \end{aligned}$$

The nodal voltages, [*V*], were calculated by inverting [*Y*] and multiplying by [*J*], as shown in Eq. (4). The difference between the signaling rail and traction rail nodal voltages on either side of the relay gave the potential difference across the relay, which was used to calculate the current through the relays.

4 $$\begin{aligned}{}[V]=[Y]^{-1}[J] \end{aligned}$$

**Table 1:** Electrical characteristics of the rails and parameters for track circuit components.

Rail Resistance	($\Omega \,\hbox {km}^{-1}$)
Signalling rail	0.0289
Traction rail	0.0289
Power supply resistor	7.2 ($\Omega$)
Relay coil resistance	20 ($\Omega$)
Pick-up current	0.081 (A)
Drop-out current	0.055 (A)

Leakage	($\text {S}\,\hbox {km}^{-1}$)
Signalling rail (wet)	0.4
Traction rail (wet)	2.0
Signalling rail (moderate)	0.1
Traction rail (moderate)	1.6
Signalling rail (dry)	0.025
Traction rail (dry)	1.530
Track Circuit Parameters	
Power supply	10 (V)
Power supply resistor	7.2 ($\Omega$)
Relay coil resistance	20 ($\Omega$)
Pick-up current	0.081 (A)
Drop-out current	0.055 (A)

### Electric field data

The modelled electric field data used in this study consist of a series of snapshot maps of the north and east components of geoelectric field computed on a $131 \times 179$ grid, each cell being approximately 10 by $10\,\hbox {km}$, covering the UK, Ireland and North Atlantic. Using the Spherical Elementary Current Systems (SECS) method^21,22^, minute-mean magnetic data from multiple observatories and variometers were used to extrapolate the field across each cell. For the March 1989 storm, four observatories and one variometer were used (Lerwick [LER], Eskdalemuir [ESK], Hartland [HAD], Wingst and Glenmore Lodge). For October and November 2003, there were nine observatories and variometers available (Valentia, Faroes, Crooktree, York, and previous). A thin-sheet modelling code was used to compute the geoelectric field from rate of change of the X and Y components of the magnetic field (dBx/dt and dBy/dt). The thin-sheet model is consists of a 1D resistivity model at depth (down to $1000\,\hbox {km}$) on top of which lies a 2D model of the surface conductance, which redistributes the induced geoelectric field along lateral contrasts in conductance, particularly coastlines or resistive geological regions. The thin-sheet code uses a single frequency (or period) so provides a strongly band-passed version of the true geoelectric field^23^. However, shorter periods of the magnetic field variation produce a larger geoelectric field response than longer periods and contain most of the power, though this depends on the local geology/conductivity. To match the Nyquist frequency of the magnetic field data (2 min), the geoelectric field was computed every 2 min (720 samples per day) which ensures the more rapid variations are captured while meeting the numerical limits of the modelling code, and also allowing a geomagnetic storm to run in a reasonable amount of time. The output of the thin-sheet code has been compared against geoelectric field measurements at HAD observatory. The magnitude and phase match reasonably well, though the model tends to underestimate the geoelectric measurements at ESK. HAD was chosen as the best site to match the geoelectric field to, as ESK, while being geographically closer to the lines being studied, is locally affected by a known mid-crustal anomaly, so is not representative of the region as a whole. For both the Glasgow to Edinburgh line and the Preston to Lancaster section of the WCML, the electric field strength for the cell that each track circuit block occupies was extracted to allow spatial variability in the track circuit model. The orientation of the rail in each block was then used alongside the north and east components of the geoelectric field to compute the parallel component along the rail. The extreme storms studied in this paper were scaled from the March 1989 storm by a factor of 2 for the 1-in-100 year storm and for the 1-in-200 year storm. This scaling was based on initial extreme value analysis of the magnetotelluric transfer (MT) functions for LER, ESK and HAD, and from results derived in Kelly et al.^24^ and Thomson et al.^25^. However, there is no clear definition of what an extreme storm return period is, so these scaling factors are only meant to loosely reflect the range of what a 1-in-100 and 1-in-200 year event could experience. In future, the geoelectric will be estimated from MT functions measured at over 75 sites in the UK which will help improve estimates of the hazard posed and to provide better local modelling of the geoelectric field.

## Data Availability

Network Rail standard documents can be obtained from https://global.ihs.com/csf_home.cfm?&csf=NR. Data used for modeling are available at https://doi.org/10.17635/lancaster/researchdata/635.
